# Supplementary material for: Are carbon emissions trading and green financial instruments synergistic? -Comprehensive quantitative research based on content analysis
Source: PLoS One. 2024 Mar 7;19(3):e0298601. doi: 10.1371/journal.pone.0298601 (PMC10919601; doi:10.1371/journal.pone.0298601)
Supplement: S1 Data — (ZIP) [file pone.0298601.s001.zip › Data/地级市Z_F工作报告.zip!/╔╜╬≈/┤≤═1⁄4╩╨/└┤╫╘╣1⁄2╓┌║┼í╛┬φ┐╦╩2╛▌═°í┐_2011.docx]

各位代表:

现在,我代表市人民政府向大会作工作报告,请各位代表审议,并请市政协委员和其他列席人员提出意见。

一、“十一五”时期经济社会发展回顾

“十一五”时期是大同发展史上极不平凡的五年,是打基础、立长远、谋未来的五年,是理思路、定战略、抓发展的五年,是化危机、破难题、促转型的五年。五年的成就来之不易,五年的经验弥足珍贵,五年的前景令人鼓舞。

一是突出转型发展,拓宽快速提升大空间。确立转型发展、绿色崛起的战略,着力推进文化名城、旅游名都、生态名邑、经济强市“三名一强”建设步伐,重力突破支柱产业单一的发展困局,开辟多元化发展的广阔前景,综合实力和竞争力显著增强。全市地区生产总值从2005年的376.51亿元增加到2010年的694.33亿元,年均增长10.53%;财政总收入从61.99亿元增加到138.42亿元,年均增长17.43%;一般预算收入从20.42亿元增加到55.17亿元,年均增长21.99%;全社会固定资产投资从145.34亿元增加到576.71亿元,累计完成1812.13亿元,年均增长31.74%;社会消费品零售总额从132.9亿元增加到303.01亿元,年均增长17.92%。

二是突出园区建设,构建跨越发展大平台。规划布局并强力推进装备制造、新医药、新材料、清洁能源、光伏产业、循环经济、冶金管材等总规划占地140平方公里的七大园区建设,打造空间集中开发、资源集约利用、产业集群发展、服务集聚配套的发展平台,招商引资呈现强劲发展势头。蓝星集团投资12亿元、年产3万吨氯丁橡胶,中国重汽投资12亿元、年产25万台变速箱,国药集团投资12亿元抗生素基地,冀东集团投资15亿元、年产500万吨水泥生产线等项目相继投产。华泰汽车、中国化工新材料、国药抗生素基地、中海油煤制天然气、陕西重汽天然气专用汽车、庞大汽车文化广场、华润啤酒等一批重大项目落地。煤电产业改造提升加速。地方煤矿由2005年的432座关闭整合重组为65座、产能4860万吨。同煤塔山2000万吨、同忻1000万吨矿井建成投产,东周窑1000万吨矿井今年投产,马道头2000万吨矿井明年投产。发电装机容量由305万千瓦增加到720万千瓦,电网供电能力由515万千伏安增加到781万千伏安。

三是突出特色城市,塑造影响全国大作品。抢占中国特色城市的制高点,精心打造名城古都、御东新区传统与现代两大城市景观,大同的凝聚力、吸引力和号召力进一步增强。全市新建改造城市道路121条、300公里,完成投资123.71亿元。御东新区道路框架基本形成,博物馆、图书馆、大剧院、体育中心等标志性工程开工建设,一医院、五医院、中医院建设主体完工,文瀛湖大景区建设进展顺利,御东、御西26个城中村改造全面推进。名城保护格局初显,东城墙、华严寺、善化寺、文庙、关帝庙、清真寺、纯阳宫、法华寺、帝君庙、华严寺广场等十大工程以独特的审美价值为“一轴双城”增添了魅力。

四是突出优质高效,发展强县富民大农业。标准化养殖小区发展到350个,其中万头以上的规模养猪场5个,奶牛存栏由4.8万头增长到7.5万头,设施农业由1万亩突破8万亩,龙头企业由80户增长到135户。华晟果蔬、绿苑饮品、雁门清高等一批农产品龙头企业发展势头强劲,从生产、加工、包装到营销实现了历史性的跨越,成为“十一五”时期农业的最大亮点。孤山水库竣工,唐河水库完成主体。新农村建设85个试点村、619个重点推进村完成“四化四改”和“五个一”工程建设任务。扶贫移民搬迁256个村、15958人。完成村通水泥(油)路8693公里,解决农村饮水安全人口84.96万人,建设村级卫生室661所,完成村通广播电视1035个村,中小学校舍改造95万平方米,全市农村基本实现“五个全覆盖”。

五是突出生态文明,创造山川秀美大环境。全市城市绿化投资22亿元,新增绿化面积1280万平方米;集中供热投资20亿元,拆除燃煤锅炉817台,新增集中供热面积3040万平方米,覆盖率达到了87%;污水处理投资5亿元,新建配套管网357公里,污水处理率由49%提高到78.2%;城市供气管网投资3亿元,气化率由75%提升到95%;供水管网投资4.19亿元,供水普及率达98%以上;环卫基础设施投入5.1亿元,城市道路机扫率达到80%;城市生活垃圾无害化处理率达到90%以上。单位生产总值能耗、二氧化硫排放量、化学需氧量均完成或超额完成目标任务。市区二级以上良好天数由220天增加到346天。创建省级园林城市目标实现。富乔垃圾电厂被授予国家级人居环境范例奖。

六是突出文化品牌,打造文化旅游大产业。以中国历史文化名城为主体,以世界遗产云冈石窟、国家风景名胜区北岳恒山为两翼,以中国大古都为龙头,打造名城古都文化、魏辽佛教文化、边塞卫戍文化、影视基地文化、艺术会展文化、夏都休闲文化六大产业。名城保护复兴工程进展顺利,云冈大景区建设成效明显,恒山综合整治稳步推进。成功举办了第八届中国民间艺术节暨第九届云冈国际文化艺术旅游节,中国大古都的历史地位被确认,中国雕塑之都成为影响深远的一张新名片。

七是突出民生改善,建设幸福大同大体系。按照“拆迁谁、改变谁、造福谁”的要求,高标准建设安置房582万平方米、65460套,总投资116亿元。就业水平稳定提高。累计新增城镇就业岗位28.26万个,下岗失业人员再就业13.57万人,城镇登记失业率3.5%,农村劳动力转移就业25.2万人。城镇居民人均可支配收入16103元,农民人均纯收入4063元,年均分别增长13.56%和10.52%。医疗保险实现全覆盖。新型农保试点县增加到5个,城乡低保对象实现应保尽保。解决市属关闭破产企业318户、近8万下岗职工的安置和善后问题,费用36亿元。教育卫生投入持续加大,财政投入累计增加67亿元。城乡统筹力度加大,总长393公里的大呼、荣乌、天大、同源、广源5条高速公路全部开工,市区一小时经济圈可望形成。人口与计划生育工作不断加强,低生育水平保持稳定。“平安大同”建设深入推进,社会治安综合治理、食品药品监管进一步加强,安全生产形势保持平稳,和谐社区建设成效明显。

八是突出自身建设,推进行政效率大提高。自觉接受人大及其常委会监督,支持政协参政议政,办理人大代表建议735件、政协委员提案1617件,办复率100%。认真听取各民主党派、工商联、无党派人士的意见,支持工会、共青团、妇联等人民团体开展工作。大力推行政务公开,深化行政审批制度改革,将市级原有的411项行政审批事项精简压缩到182项,审批法定时限缩短二分之一。积极稳妥推进事业单位清理规范和分类改革,撤销23个、合并45个、转制7个。建立完善并严格执行规划管理、工程招投标、土地出让、财政评审、国企破产、人事管理等制度,政府管理逐步纳入制度化轨道。行政事业单位坚持凡进必考,严把进人关,优选高素质专业人才。坚持一线工作法,发扬“三事”精神,政府执行力和公信力进一步提高。

所有这些成绩的取得,归功于省委、省政府和市委的正确领导,归功于全市广大干部群众的团结拼搏,归功于各民主党派、工商联、人民团体和各界人士的广泛参与,归功于驻同中央和省级机关、企事业单位、人民解放军、武警官兵的大力支持。在此,我代表市人民政府,向所有关心、支持和参与大同改革发展的同志们、朋友们,表示衷心的感谢和崇高的敬意!

与此同时,我们也清醒地看到,我市经济社会发展中的短期问题和长期问题交织,结构性问题和体制性问题并存,经济总量不大、结构不优,加快转型跨越发展迫在眉睫;城乡差距仍然较大,县域经济发展滞后,贫困县比例较大,强县富民任重道远;名城保护与新区建设任务艰巨,拆迁安置面广量大,处理好发展与稳定的关系面临严峻挑战;城市管理、社会管理的体制机制尚未理顺,管理粗放的问题比较突出,与规范化、制度化、科学化的目标差距甚远;人才队伍结构性矛盾突出,专业化、知识化、年轻化进程缓慢,远远不适应改革发展的要求;服务型政府建设存在差距,服务水平和工作效率需进一步提高,党风和廉政建设需进一步加强。这些矛盾和问题事关全局,影响长远,需要我们采取更加有力的措施,努力加以克服和解决。

二、“十二五”时期的奋斗目标和主要任务

按照《中共大同市委关于制定国民经济和社会发展第十二个五年规划的建议》,我们编制了《大同市国民经济和社会发展第十二个五年规划纲要(草案)》,提交大会审议。

“十二五”是决定大同未来的关键时期。牢牢抓住历史的机遇,顺应人民群众过上更好生活的新期待,抢占竞争的制高点,打好文化牌;抢占发展的制高点,走出转型路;抢占战略的制高点,创造特色城,再造一个新大同,是历史赋予我们的使命,是人民群众的重托和期待。“不谋万世者不足谋一时,不谋全局者不足谋一域”。认清大势方能做成大事,我们面对的机遇和优势前所未有,面临的挑战和风险前所未有,必须以忍辱负重的博大胸怀,精卫填海的牺牲精神,毁誉由人的达观态度,壮士断腕的坚强决心,不计个人得失进退,义无反顾,勇往直前。

(一)抢占竞争制高点,打好文化牌

当代国际经济技术军事竞争,起决定性作用的是文化竞争。文化强国才是真正的强国。文化已成为国际竞争的核心要素,一个城市综合竞争力提升的核心引擎。文化承载着历史,传承着文明,以其强大的历史穿透力、文明渗透力、艺术感染力、民族凝聚力,发挥着人类精神之魂的独特作用。文化是城市的名片、城市的记忆、城市的内涵、城市的形象和品位。几乎所有的城市都是靠文化来立世和扬名的,没有文化的城市等于没有灵魂,永远不可能成为世界名城。

大同具有丰富的优质文化资源,“三代京华,两朝重镇”的辉煌历史,给大同留下丰厚的城市记忆。中国历史文化名城、世界遗产云冈石窟、国家风景名胜区北岳恒山、中国大古都就是城市记忆中国宝级的华章巨篇。这在传统文化遭遇建设性破坏,众多历史文化名城有历史却没有记忆,失去了体味历史权利的今天,显得尤为珍贵。记忆是城市的灵魂,留住了城市记忆就是留住了城市历史,这是城市文化建设的基础,是大同打好文化牌的核心所在。要始终不渝坚持“最少干预、整旧如旧”的原则,按照原形制、原结构、原材料、原工艺“四原”的方法,精心做好文化遗产保护修复工程,最大限度保存一切历史信息和文化记忆,让大同文化以完整性、真实性的沧桑厚重之美,稀世特立,光照寰宇。“十二五”时期,大同要将古城、云冈石窟、北岳恒山、大古都四篇文章做大做强,做精做优,具有中国气派,中国品牌,大同特色,大同风格,使文化资源优势真正转化为文化产业优势。

打好文化牌,还要在传承历史文化的基础上进行文化创新,大力发展文化创意产业。用创意激活地方文化,用文化构建城市魅力,用魅力吸引高端消费要素和生产要素,形成集创意产生、创意制造、创意消费于一体的产业链。积极探索“文化+科技”、“文化+旅游”、“文化+金融”等发展新模式,引进一批国内外文化创意先锋企业,引领产业升级,用高新技术改造传统文化产业,做大、做强、做优一批文化产业发展主体,打开文化产业发展的广阔空间。要以多样化的社会网络架构便捷化的创意通道,以多元化的文化体验重构开放式的创意平台,以多赢化的公共政策营造高品质的创意环境,打造强大的创意磁场,集聚国内外的英才俊杰落户创业,与华强、华侨城等十大文化创意集团联手创造文化与科技结合的主题公园,吸引更多国内外的消费者观光体验。要集聚创意人才力量创造城市文化主题,持续不断进行文化艺术会展、文化艺术会演、文化艺术博览,使大同真正成为闻名世界的文化之城、艺术之都、创意之市、朝圣之地、休闲之所。

胜在竞争力。依托文化优质资源,打造中国一流的文化产业,抢占文化产业发展的制高点,不仅关系到文化自身的振兴和繁荣,而且决定着大同未来的前途命运。城市以文化论输赢。“十二五”时期,我们要以文化重大事件来制造创意城市的磁铁聚集效应,持续不断进行新视野、新层面、新领域的探索,在文化产业发展上赢得主动,赢得先机,赢得优势。

(二)抢占发展制高点,走出转型路

后国际金融危机时期,世界经济发展格局正在发生深刻变革,培育发展战略性新兴产业,已经成为世界各国抢占新一轮经济科技竞争制高点的战略选择。我们必须登高望远,强化国际战略思维,积极应对国际竞争。要以科学发展为主题,转型发展为主线,走资源节约、环境友好、低碳发展的创新之路,发展结构优化、技术先进、清洁安全、附加值高、具有大同优势的现代产业体系。特别是在新能源、清洁能源等战略性新兴产业方面,要以全球化的战略视野,整合全球科技人才资源,加强技术创新,建设世界一流的新兴产业基地,走出一条黑色能源向绿色能源转移的可持续发展之路。“十二五”时期,要抓好七大产业基地。

新能源产业基地。新能源产业是国民经济战略性、先导性产业。未来国家新能源开发利用的战略目标是,到2020年,太阳能、风能等新兴能源要成为能源总需求中增量部分的主力军,占一次能源消费总量的15%;到2030年,新兴能源要成为主流能源之一,占一次能源消费总量的20%以上。大同作为一个传统能源基地,要充分发挥光、风资源丰富的优势,资源与技术引领,实业与金融结合,大力度推进风光储输一体化产业链,建设千万千瓦级风光储输产业基地。引进世界500强富士康、世界“硅王”保利协鑫等战略高端企业,投资1000亿元,建设10万吨多晶硅、6G太阳能电池组件,并利用大同光资源丰富的优势,延伸发展1000万千瓦太阳能电厂,形成世界最大的太阳能基地。保利协鑫在西门子改良法的基础上,利用全球高端人才资源优势,进行18项重大技术创新,形成具有世界一流水平的“GCL多晶硅生产法”,以超低的生产成本、超低的能耗、超低的建设周期,成为世界最具竞争力的太阳能企业。与世界制造业之神富士康战略合作,强强联手,将会改变世界格局,带来全球太阳能市场重新洗牌。今年1月,在胡锦涛主席对美国进行国事访问期间,国家能源局、北京大学与美国能源部签署了《太阳能十项全能竞赛合作协议谅解备忘录》,将在美国本土和欧洲成功举办了五次、具有世界最高水准的太阳能竞赛落户中国。“十项全能”竞赛,具有世博会的形式,有设计建设时间长、可永久参观和体验的特点。我市已争取列为备选城市之一,力争成为举办2013年全能竞赛的城市。在中国历史文化名城的头上,再戴一顶世界太阳能之城的桂冠。我市有丰富的风能资源,要引进远景能源等新兴能源企业,充分利用和挖掘大同的风能资源,使5米以上的风能得到高效利用,到2020年规划建设1000万千瓦风电项目,形成风光储输一体化产业链。

清洁能源产业基地。大同作为闻名全国的煤都,以传统高碳排放的黑色能源经济为主体。发展以“科学、绿色、低碳”为特征的清洁能源,构建安全、稳定、经济、清洁的现代能源产业体系,是大同转型发展的标志性工程。要紧紧抓住中海油总投资1000亿、年产100亿立方煤制天然气这项重大工程,充分利用世界500强整合全球资源的能力,做成世界一流的现代能源示范工程,使大同传统能源涅槃再生。煤制天然气兼有产业链条长的特点,可形成以煤基清洁能源为核心的现代煤化工系列产业,市场前景广阔,延伸开来就是一座化工城。

装备制造产业基地。这是大同工业的基本代表。重点突破轿车、重卡等整车制造业,提升大同制造的整体水平。建设汽车变速箱、发动机、桥齿等汽车零部件基地,形成产业集聚配套的现代制造业体系。做大做强电力机车,带动高速铁路装备制造产业高端化、系列化、成套化发展。实施开放引进的品牌战略,与国内外高端企业合作,高起点发展煤炭装备制造业。

现代医药产业基地。加强与中国医药集团深度和广度合作,建设中国医药最大的抗生素基地,大力发展固体、液体制剂,发展疫苗等生物制药产业。强力实施“同药战略”,推动优质医药企业进入资本市场,募集资金,加速发展。

新型化工产业基地。加强与中国化工集团战略合作,重点发展氯丁橡胶、TDI、MDI、ADI等新型化工材料工业,提升山西化工产业的整体水平。推进同煤甲醇、聚甲醛、烯烃等煤化工项目建设。

钢铁管材产业基地。推进太钢与同钢战略重组,建设山西北部钢铁基地。

现代服务业基地。以文化旅游产业为龙头,引进奥特莱斯、华南城等高端服务业旗舰企业,加快东小城、云中商城等区域性商业中心开发,大力发展高、中端星级酒店等服务产业,打造晋冀蒙区域现代服务业中心,融入首都现代服务产业圈。

成在发展。七大基地建成之日,就是大同转型发展实现之时。“十二五”时期,转型发展关乎大同兴衰存亡,成败在此一举。要倾其所有,竭其所能,全力以赴,务求必胜。

(三)抢占战略制高点,创造特色城

当前,我国城市发展存在严重的特色危机,表现为城市建设利益化、城市文化粗鄙化、城市景观浅薄化、城市形象趋同化、城市历史虚拟化、城市消费奢侈化、城市休闲低俗化、城市娱乐商品化。大同要从激烈的城市竞争中脱颖而出,先声夺人,不被平庸的浪潮所淹没,必须实施以特领先、以特取胜的战略,以差异化发展为目标,以专业化发展为手段,以个性化发展为核心,以特质化发展为品牌,抢占特色城市的制高点。城市是一个“磁体”与“容器”的结合体,所谓“磁体”就是吸引资源的功能,“容器”就是汇聚和保持资源、综合创新的功能。特色就是一个城市的“磁体”,就是一个城市的吸引力、号召力和凝聚力。没有特色的城市是苍白的,就会被激烈的市场竞争所抛弃。“十二五”时期,创造中国特色城市,是大同历史性的使命和城市宣言。

大同城市特色的核心和精髓是“一轴双城”。一座饱经千年沧桑的完整古城,拥有典雅、崇高的美学价值,延续着曾经的辉煌,传递着历史的回响;一座充满创造活力的现代新区,拥有新潮、大美的时代品格,熔铸着当代的精神,塑造着城市的形象。大同在历史的迷雾中找到了属于自己的文化坐标,“一轴双城”作为大同特色城市文化支点,以历史的力量为城市加固,以文明的名义为城市输氧,以艺术的经典使城市夺目,以支点的伟力使城市动容。这个文化支点是有亲和力的,它吸引成千上万的观众为之倾倒;这个文化支点是有品牌效应的,它以永恒的历史价值让城市光艳生辉、名声远播;这个文化支点是有生命的,它以不朽的存在给人们以精神的能量、理想的养分。

赢在特色。“十二五”时期,“一轴双城”建设要完美收官。3.28平方公里的古城整体恢复历史风貌,40平方公里的新区基本建成,珠联璧合,双城并美。有特色的城市才是美的城市。在大同城市的巨大容器里,装的是两大景观,一个现代景观,一个历史景观。两者形成强烈的对比和文化张力,铸就城市独特的魅力。

三、2011年工作安排

2011年是“十二五”的开局之年。做好今年的工作,对于推动转型、实现跨越起着至关重要的作用。

今年全市经济社会发展的主要预期目标是:地区生产总值增长13%;财政总收入增长15.6%;一般预算收入增长15%;全社会固定资产投资增长25%;城镇居民人均可支配收入增长10%;农民人均纯收入增长10%;居民消费价格总水平涨幅控制在4%左右;城镇登记失业率控制在4%以内;人口自然增长率控制在6.5‰以内。

主要约束性指标是:万元生产总值综合能耗下降3.5%左右,万元生产总值二氧化碳排放量下降3.5%,二氧化硫、化学需氧量排放量分别下降2.36%和2%,氮氧化物下降2.19%,氨氮下降1.83%,烟尘、粉尘各下降2%,万元工业增加值用水量下降5%。安全生产形势明显好转,坚决遏制重特大事故发生。

实现上述目标,重点要抓好以下几方面工作:

(一)以园区建设为平台,以招商引资为抓手,强力推进转型跨越发展

高标准、高起点建设好七大园区,富集生产要素,完善配套功能,构筑发展平台。装备制造园区要抓紧20个已落地项目的开工建设,尽快形成现实生产能力。特别是要抓好陕汽重卡、大齿二期、华润啤酒、玄武岩连续纤维等重大项目建设。医药园区要继续推进中国医药集团战略重组,加快抗生素基地建设,向年销售100亿冲刺。加快普德、仟源、泰盛、同达等医药企业入园建设,实现规模和技术整体创新。光伏产业园区要加快基础设施建设,开工建设由富士康和保利协鑫共同投资总规模402亿元的太阳能完整产业链,其中多晶硅5万吨／年,投资210亿元,配套电厂2×60万千瓦,投资52亿元,太阳能电池组件3吉瓦、起步300兆瓦,投资110亿元,太阳能电站起步200兆瓦,投资30亿元,初步构建太阳能光伏产业链框架。塔山循环园区要加快完善路网及配套功能,推进塔山220千伏变电站建设,开工建设2×30万千瓦热电联供电厂,拓展延伸循环产业链。龙泉化工园区要抓紧氯丁橡胶二期、13万吨TDI、50万吨电石等项目建设。左云清洁能源园区要启动基础设施建设,为40亿立方煤制天然气开工创造条件。灵丘冶金园区300万吨钢铁项目要按照关小上大、淘汰落后、资源整合的原则,加快前期准备工作,力争今年开工建设。

招商引资是转型跨越发展的第一推动力。招来商、引来资,才有项目;有项目、有投入,才有发展,这是最基本的道理。今天的大同,招商引资是转型跨越发展的唯一希望。以发展论英雄,招商引资是第一能力、第一政绩。市县党政领导干部都要拿出极大的精力抓招商引资,不断提高招大商、引大资的能力。招商引资,第一是信息,知道商在哪里,捷足先登,抢占先机;第二是诚信,万事以诚为本,以信为先,诚信是金不换的招牌;第三是坚持,顽固一点,扭住不放,坚持到底;第四是效率,说办就办,马上办,办得好;第五是舍得,舍得辛苦,舍得付出,舍得求人,舍得放下架子,有舍才有得。有了这十个字精神,成功就在眼前。

要抓住“综改试验区”的机遇,敢闯敢冒,先行先试,争取享受更多的政策红利。要紧紧盯住中海油、富士康、保利协鑫、中国医药、中国化工、中国重汽、中国建材、华润、华泰、华能、台湾华宇、陕西重汽、冀东物流等大集团、大企业,一个项目一个项目落实,提高招商引资的有效性。

要加快传统产业的改造提升,全力推进煤炭企业兼并重组后的矿井改造。今年是矿井建设年,开工率必须达到100%,产能达到设计能力的50%,全市煤炭总产突破1.1亿吨。

要加快电网基础设施建设,重点推进500千伏大同东、220千伏塔山、左云马道头等重点项目建设。

(二)以“一村一品、一县一业”为载体,以龙头骨干企业为支撑,提升县域经济的综合实力

再造一个新大同,关键在县域经济。县域经济总量偏小、后劲偏弱,是大同经济发展的最大“软肋”。振兴县域经济,是大同转型跨越发展的重大战略。在工业化、城镇化进程中推进农业现代化,按照产业集群、技术集成、资本积聚的要求,加快17个农业示范园区的建设进程。要实施“一村一品、一县一业”的发展方略。南郊的蔬菜和养牛业、左云的马铃薯、阳高的大棚蔬菜和养猪业、浑源的黄芪、大同的黄花菜、广灵的菌类和杂粮、天镇的玉米、灵丘的干果、新荣的肉羊养殖和马铃薯,初显“一县一业”的发展格局。抓住重点,强力扶持,小中见大,长期坚持,就能做出一片天地来。

龙头骨干企业是县域经济的支撑。要重点扶持10个旗舰型、20个潜力型、30个成长型龙头企业,为县域经济发展增添新的动力。要抓好雨润百万头生猪屠宰、华晟果蔬小包装生产线、四方高科5000头奶牛、承德露露与绿苑饮品合作的软包装生产线,力争有两户农产品龙头企业年收入突破50亿元、10亿元,推动农业产业化龙头企业规模化、优质化、品牌化。

设施农业是富民产业,是大同农业产业化的必由之路。要坚定不移强力推进,朝着户均一亩大棚的目标奋斗。要继续采取以奖代补政策,今年新建日光温室、塑料大棚、移动大棚3万亩,总量突破10万亩。要加快推进标准化健康养殖小区建设,今年建设50个示范区,不断提升农业产业化水平。要引进北京新发地等特大型农产品交易市场,建立覆盖全国的农产品交易平台,扩大大同农产品销售的市场半径,推动农业产业化的深度发展。农业最终要靠科技解决问题。要加强农业科技推广和服务体系建设,帮助农民解决产前、产中、产后面临的实际困难,提高科技服务对农业产业化的贡献率。

加强水利基础建设。加快建设墙框堡水库和引黄供水工程,实现国庆节引黄河水至大同目标。完成唐河水库建设工程,开工建设守口堡水库。进一步改善和提高15万农村人口的供水质量和标准。完成御河、口泉河河道综合治理工程。新增节水灌溉面积10万亩,改善恢复灌溉面积10万亩。要充分发挥引黄工程的效能,高标准规划建设百万亩水浇地,从根本上改变农业生产的基础条件。加快实施新一轮农网改造升级工程。

加快新农村建设,推进城乡服务一体化。巩固试点村和重点推进村的新农村建设成果;完成新确定的重点推进村建设任务,完成市级新农村建设规划,扶持建设18个新农村样板,选择1-2个县开展城乡一体化建设试点,进行集中连片建设。在两年内完成农村街巷硬化、农村便民连锁商店、农村文化体育场所、农村职业教育免费、新型农村社会养老保险“五个全覆盖”工程。完成3500人扶贫移民搬迁任务,启动阳高、浑源、广灵片区扶贫开发项目。

(三)以古城保护为主体,以云冈完善和恒山整治为两翼,加快文化旅游产业崛起

加快“一主两翼”建设步伐,推动文化旅游大产业发展。文化名城保护修复要完成北城墙、代王府、鼓楼东西历史街区、北魏明堂公园、法华寺、南城墙瓮城修复等工程,完成文庙、关帝庙环境整治及华严寺、清真寺商业广场、法华寺商业街区建设工程。云冈石窟要加快推进窟檐改造和防渗工程,完成云冈数字化陈列馆、演艺中心主题表演等项目,举办好云冈盛大的佛事活动。北岳恒山和悬空寺要加快景区建设及环境整治工程,完成停旨岭村的搬迁及村庄绿化,岳门湾景区、出入口景观建设以及神溪湿地、文庙、圆觉寺和历史街区的修复工程。加快晋华宫国家矿山公园、煤气公司工业遗址公园建设工程。积极推进丝绸之路联合申遗工作。建设中国民间文化艺术博物馆和雕塑艺术馆,推进福建隆德大同论坛会址、御东会展中心、影视基地等一批文化产业项目。举办好民间文化艺术节、旅游节和雕塑、美术双年展等节庆活动。创作以北魏王朝为题材的电视连续剧,打造高水平的旅游文化主题表演,大品牌拉动,大平台推动,大文化引领,进一步强化宣传大同、营销大同的力度,提升大同的知名度和影响力。要下大力引进华强集团,建设全国一流创意文化产业园区,激活传统文化要素,让创意文化与传统文化比翼齐飞,相得益彰,共创大同世界旅游终极目的地的美好未来。

发展文化旅游大产业,政府创造环境,企业经营文化,社会参与创造,全民共享成果。一个城市在精神的世界里强大,才会在现实的世界里强大。全市人民都要以高度的文化自觉和主人翁责任感,共同创造城市道德气候和城市精神,广泛参与文化旅游产业创造,让大同浓郁的地方活态文化成为旅游城市一道靓丽的风景线,为文化旅游产业大发展不断注入人文活力。

(四)以道路建设为重点,以生态绿化为亮点,继续推进城市基础建设

今年是城市建设在“一年一个样,三年大变样”基础上,实现“五年变新样”的关键之年,必须继续加大投入,强化特色,全力推进,实现城市根本创新。道路、边缘、区域、节点和标志,是城市建设的五大要素。道路是一个纵向展开的城市景观,又是城市流动的血脉。要抓好御东、御西和园区60条、90公里道路改造建设,让城市生活更美好。边缘是城市平面和立体的外在形象。要继续推进城市道路两侧立面改造,让具有大同特色的文化元素融入街巷、社区,规划控制好城市的天际线。区域是城市内部展开的景观。要抓好东小城广场、南瓮城广场、御东文化中心广场等三大中心区域,展示城市的风采和魅力。节点是城市交汇的重点穴位。要抓好道路、河流交叉点系统性建设,让城市更通畅、更靓丽。标志是城市的图腾、城市的记忆、城市的形象符号。古城墙是大同历史文化名城的标志,博物馆、美术馆、图书馆、大剧院、体育中心等五大建筑是御东新区的标志,要精心打造,真正成为撼人心肺的大作品。

生态是城市的生命。要实施好文瀛湖景观、御河蓄水、十里河湿地、南城墙公园、采凉山森林公园等14项大型生态工程,向国家园林城市的目标进军。要强力推进城乡植树造林,高标准迎接明年全省造林绿化大同现场会。继续推进生态工程治理,加强环境保护。集中供热、污水处理、供气、供水继续推进全覆盖,开展节能减排,加强源头治理,为实现蓝天、碧水、绿地、宁静、洁净的目标打好基础,确保完成省级环保模范城市创建工作。

管理是城市永恒的主题。继续推进清扫机械化、收集容器化、清运密闭化、保洁全天化,夯实卫生大同的基础。进一步完善“两级政府、三级管理、四级网络”的管理格局,强化区级政府管理城市的主体功能。发挥好数字化管理平台的功能,建立发现及时、处置快速、解决有效、监督有力的长效管理机制,使城市管理步入规范化、制度化、科学化、经常化的轨道。要强化全民“爱我大同、净化大同”的意识,营造城市管理创新的环境和氛围。

要强化以城带乡、城乡统筹。完成得大高速公路改线工程,加快荣乌、天大、同源、广源高速公路建设进度,今年完成200公里。完成国道208一级公路改建工程。加快大同客运东站前期工作。开工大同——张家口(大同段)、大同——西安(大同段)高速客运专线建设工程。完成大同机场改扩建工程。

(五)以保障改善民生为目标,以为民多办实事为着力点,推进幸福大同建设

以人为本,执政为民,让人民群众生活得更幸福、更有尊严,是我们党的本质属性,是政府的职责使命所在,是我们一切工作的出发点和落脚点。民评民说是标准,民心民力是依靠,民意民声是依据,民愿民盼是方向,民惠民富是目标,民苦民痛是失职。

加快拆迁安置保障住房建设。城市棚户区改造和保障性住房新开工建设222.35万平方米、23286套,续建完工356.25万平方米、39609套。今年及以前的拆迁户要实现百分之百的安置。继续推进同煤“两区”改造安置房建设,新开工234.8万平方米、17704套,续建296.3万平方米、28730套。要坚持规划建设管理的高标准,真正把安置工程办成民心、民有、民富、民和、民乐工程,让人民群众得到真正的实惠。

积极扩大就业。坚持就业数量增长与质量提升并重,多渠道开发就业岗位,最大限度增加就业容量。大力推进创业培训工作,提高创业本领,以创业带动就业。进一步落实就业扶持政策,不断提高就业服务质量和服务效率。继续做好就业困难人群中特殊群体的就业工作,特别是高校毕业生和军队退役人员的就业安置工作。

完善社会保障体系。继续坚持“广覆盖、保基本、多层次、可持续”的方针,扩大社会保险覆盖范围。重点做好失地农民、非公有制经济组织就业人员、城镇灵活就业人员参加社会保险,保持各项社会保险覆盖人数和基金收入的同步增长。进一步提高城乡居民最低生活保障标准,切实做到动态管理、应保尽保。继续加快养老服务等社会福利机构建设,健全和完善社会救助体系。

加强教育基础建设。扎实推进义务教育标准化建设,继续巩固“普九”成果,使广大人民群众子女共享优质教育资源。关注职业教育发展,努力扩大职业高中招生规模。实施好大同五中、九中、十二中、十三中、十六中、实验小学等学校的新建工程。进一步加大筹资力度,加快工程进度,狠抓工程质量,完成中小学校舍安全工程建设任务。

扩大优质医疗资源。完成市一医院、五医院、中医院、六医院新建工程。健全基层医疗卫生服务体系,抓紧实施11个县级医院、乡镇卫生院、社区卫生服务中心的项目建设。全面实施基本药物制度,加快公立医院改革。推进住院费用支付方式改革,控制医药费用的不合理增长。重新修订区域卫生规划和医疗机构设置规划,科学合理整合卫生资源。坚持计划生育的基本国策,稳定低生育水平,提高出生人口素质。

做好安全稳定工作,促进安全生产形势的持续稳定好转。完善信访工作机制,从源头上预防和化解社会矛盾。积极探索安民、便民、惠民的社会管理和服务体系,大力提升城市现代化管理水平。加强流动人口服务和管理,进一步做好民族和宗教工作,深化“双拥”共建,努力开创全市人民各尽其能、和睦相处的良好局面。

(六)以提高效率为中心,以优质服务为标尺,加强政府自身建设

办好大同的事,实现“十二五”时期再造一个新大同的宏伟目标,政府必须是一个为民、廉洁、高效的好政府。

要提高政府的公信力。公信力是政府的立身之本。公信就是公正、廉明、诚实、信用。政府想问题、作决定、办事情,都要出以公心,“为天地立心”,维护公平正义,和人民群众坐在一条板凳上,做到“威武不能屈,富贵不能淫,贫贱不能移”,代表最广大人民的根本利益。要廉洁操守,谨慎用权、公正用权、廉洁用权,“情为民所系,利为民所谋,权为民所用”。要不断加强政府廉政建设,做到敢干事、会干事、干好事、不出事。政府拥有公权,切忌公权私用。“心底无私天地宽”。政府要诚实守信,说话算数,不打折扣,决不能“口言惠而实不至”。政府公信力源于公众的信任,信任的重要前提就是了解。只有保障人民的知情权、参与权、表达权、监督权,确保权力在阳光下运行,政府公信力才能伴随阳光不断生长。

要提高政府的执行力。执行力是政府的生命之基。赢在执行。一个行动胜过一打纲领。执行力的核心在效率,效率的概念是个时间概念。时间的浪费是最大的浪费。在规定的时间内,高质量办好应该办的事,就是效率。要在政府培养马上办、办得好、快速高效的政风,克服“不想干、不会干、不敢干”的惰性。要创造干事创业的氛围,让想干事的有机会,能干事的有舞台,干成事的有地位。要提倡抓具体、抓落实、抓实效的良好风气,把办公桌搬到第一线,任务在一线完成,问题在一线解决,业绩在一线创造,成效在一线检验。

要提高政府的亲和力。亲和力是政府的力量之源。要把群众呼声作为第一信号,把群众需要作为第一选择,把群众满意作为第一标准,多办顺民意、解民忧、增民利的实事。榆次县衙有一副对联:“吃百姓之饭,穿百姓之衣,莫道百姓可欺,自己也是百姓;得一官不荣,失一官不辱,勿说一官无用,地方全靠一官”。古人尚知如此,我们更应该与人民群众打成一片,树立亲民政府形象,从人民群众那里汲取智慧和力量。

各位代表,“十二五”的序幕已经拉开,宏伟的目标激动人心,美好的前景催人奋进。让我们在省委、省政府和市委的领导下,紧紧抓住历史机遇,勇敢面对各种挑战,打好文化牌,走出转型路,创造特色城,为实现再造一个新大同的宏伟目标而努力奋斗!
